# Towards Clinical Application of Neurotrophic Factors to the Auditory Nerve; Assessment of Safety and Efficacy by a Systematic Review of Neurotrophic Treatments in Humans

**DOI:** 10.3390/ijms17121981

**Published:** 2016-11-26

**Authors:** Aren Bezdjian, Véronique J. C. Kraaijenga, Dyan Ramekers, Huib Versnel, Hans G. X. M. Thomeer, Sjaak F. L. Klis, Wilko Grolman

**Affiliations:** Department of Otorhinolaryngology and Head & Neck Surgery, Brain Center Rudolf Magnus, University Medical Center Utrecht, 3584 CX Utrecht, The Netherlands; aren.bezdjian@mail.mcgill.ca (A.B.); V.J.C.Kraaijenga@umcutrecht.nl (V.J.C.K.); D.Ramekers@umcutrecht.nl (D.R.); H.G.X.M.Thomeer@umcutrecht.nl (H.G.X.M.T.); S.Klis@umcutrecht.nl (S.F.L.K.); W.Grolman@umcutrecht.nl (W.G.)

**Keywords:** neurotrophin, growth factor, clinical trial, neurodegenerative disorders, inner ear, peripheral nervous system, translational medicine

## Abstract

Animal studies have evidenced protection of the auditory nerve by exogenous neurotrophic factors. In order to assess clinical applicability of neurotrophic treatment of the auditory nerve, the safety and efficacy of neurotrophic therapies in various human disorders were systematically reviewed. Outcomes of our literature search included disorder, neurotrophic factor, administration route, therapeutic outcome, and adverse event. From 2103 articles retrieved, 20 randomized controlled trials including 3974 patients were selected. Amyotrophic lateral sclerosis (53%) was the most frequently reported indication for neurotrophic therapy followed by diabetic polyneuropathy (28%). Ciliary neurotrophic factor (50%), nerve growth factor (24%) and insulin-like growth factor (21%) were most often used. Injection site reaction was a frequently occurring adverse event (61%) followed by asthenia (24%) and gastrointestinal disturbances (20%). Eighteen out of 20 trials deemed neurotrophic therapy to be safe, and six out of 17 studies concluded the neurotrophic therapy to be effective. Positive outcomes were generally small or contradicted by other studies. Most non-neurodegenerative diseases treated by targeted deliveries of neurotrophic factors were considered safe and effective. Hence, since local delivery to the cochlea is feasible, translation from animal studies to human trials in treating auditory nerve degeneration seems promising.

## 1. Introduction

Sensorineural hearing loss is the most common form of hearing loss that encompasses pathologies of the inner ear and the auditory nerve. Cochlear implantation, the current therapy for profound sensorineural hearing loss, is known to be contingent on the presence of spiral ganglion cells and function of the auditory nerve [1]. The ongoing degeneration of the auditory nerve that occurs following sensorineural hearing loss is considered a limiting factor in cochlear implant efficacy [2,3,4]. A reduced number of auditory nerve fibers in the human ear also underlies pertinent disorders such as hidden hearing loss [5,6].

A class of secreted proteins called neurotrophic factors (NFs) has shown to play a key role in the survival of the auditory nerve [5,7,8]. NFs are essential during the development and differentiation of the central nervous system (CNS), a time when specific synaptic connections and circuits are being formed. Later, in the mammalian adulthood, neurotrophin signaling plays an important role in the continued maintenance and modulation of those connections required for optimal brain function [9,10]. Since their discovery in the 1950s by Levi-Montalcini [11], a discovery worthy of a Nobel Prize in medicine, in vitro and in vivo animal experiments have elucidated their strong ability to elicit positive survival and functional responses in neurons of the peripheral and CNS [12]. Consequently, these findings rendered NF proteins as ideal drug candidates for the treatment of various disorders due to their abilities to promote neuronal survival, to stimulate cell function and thus, alter disease progression. Since to date none of the NFs have FDA approval, robust pre-clinical and clinical studies are essential.

A suitable target for treatment with NF proteins could well be the mammalian ear. Exogenous delivery of NFs into the cochlea of deafened animals has continually demonstrated beneficial functional and morphological preservation of the auditory nerve [4,13,14,15,16,17,18,19]. Based on these preclinical studies, it is hypothesized that NF therapy may provide beneficial effects and improve cochlear implant performance or prevent long-term neural loss after noise exposure [4,19]. However, this has not yet been clinically investigated due to safety and applicability concerns. Given the patency of the cochlear aqueduct with the cerebrospinal fluid, active agents delivered to the human cochlea have the potential to enter the brain and elicit effects throughout the CNS, which may lead to adverse events (AEs) [20].

In the past two decades, the translation from animal studies to human clinical trials involving NFs has been made with respect to various degenerative disorders. Scientific evidence in in vivo animal models revealed promising results to treat neurodegenerative diseases such as amyotrophic lateral sclerosis (ALS) and Parkinson’s disease. For instance, systemic ciliary neurotrophic factor (CNTF) treatment protected motor neurons and improved motor function in mouse models characterizing motor neuron deficits such as in ALS [21,22]. Intracerebral treatment with glial cell line-derived neurotrophic factor (GDNF) in rodents and monkeys with Parkinson’s symptoms demonstrated an increase of dopamine levels and improvement of motoric behavior [23,24]. The promising outcomes in animal models [25,26] led to multicenter clinical trials [27,28,29,30,31].

Safety and efficacy data provided by various clinical trials using NFs could provide invaluable input in setting up desired clinical trials to treat auditory nerve degeneration. Although there is large heterogeneity in the available literature, available data from past trials using NFs may nonetheless provide useful directions for the applicability of NFs to the auditory nerve. Therefore, in order to address the safety and efficacy concerns of NF therapies in clinical settings, we have conducted a systematic review of the best available evidence concerning NF treatment in humans.

## 2. Results

### 2.1. Search and Selection

**Figure 1:**
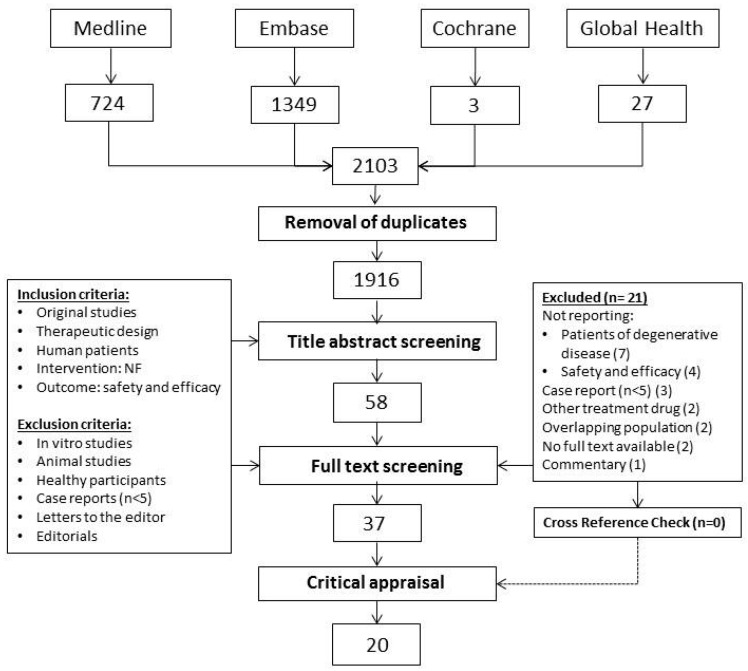
Flow chart demonstrating study selection process.

A total of 2103 articles were retrieved from the database searches. From these, 1916 unique articles were screened for title and abstracts. Following selection based on titles and abstracts, 37 articles were chosen for full text review. Searching clinicaltrials.gov yielded two additional studies that were not published: a phase 1 study on continuously infused intracerebral (IC) recombinant-methionyl human GDNF (r-metHuGDNF) for the treatment of idiopathic Parkinson’s disease (NCT00006488) and continuously infused r-metHuGDNF to treat progressive supranuclear palsy (NCT00005903). Cross-reference examination yielded no additional relevant articles. For two study population more than one article reported results from the same cohort [29,30,32,33,34]. Therefore, a total of 37 articles describing 34 unique human trials were considered for quality assessment (Figure 1).

### 2.2. Critical Appraisal Assessment

Studies were assessed for their directness of evidence (DoE) and risk of bias (RoB). The DoE was high in 18, moderate in 16 and low in two studies. Twenty articles successfully passed the critical appraisal and were included for data extraction, of which 12 scored high for DoE and low for RoB, and eight scored moderate for DoE and low for RoB (Table 1 and Table 2). All 20 included studies were randomized controlled trials (RCTs) published between 1995 and 2015.

### 2.3. Patient Characteristics and Treatment Modalities in Selected Studies

Patient demographics and outcome data on treatment modalities extracted from the 20 RCTs [27,28,31,35,36,37,38,39,40,41,42,43,44,45,46,47,48,49,50,51] included in the present review are reported in Table 3 and summarized in Table 4. A total of 2445 patients were treated with a NF, while the remaining 1529 patients were assigned to the placebo group. The mean age of included patients receiving NF therapies was 55.2 (±10.4) years. No pediatric patients were included in any of the trials. ALS was the most frequent diagnosis investigated in 2090 patients (53%), followed by diabetic polyneuropathy in 1113 patients (28%), retinitis pigmentosa in 266 patients (7%), obesity in 173 patients (4%), sudden deafness in 118 patients (3%) and Parkinson’s disease in 84 patients (2%). CNTF was the most frequently used NF drug in 1219 patients (50%), followed by NGF in 580 patients (24%) and IGF-I in 510 patients (21%). The study drug or placebo was administered systemically in 3409 patients (86%). The remaining patients (*n* = 565, 14%) received targeted local therapies. Duration of treatment was more than six months for 2669 patients (67%), between one to six months for 1082 patients (27%), less than one month for 105 patients (3%) and was not known for the remaining 118 patients (3%). Note that pooling of the available evidence for subsequent meta-analysis was not possible as a result of the inevitable heterogeneity of the studies.

### 2.4. Safety Assessment and Adverse Events Reported in Selected Studies

Although AEs during and after treatment were investigated in all studies, two studies did not report quantifiable data. Therefore, Table 5 does not include the patients receiving NF treatment in these studies (*n* = 485 and *n* = 124; respectively) [27,35]. One of these RCTs was one of the two articles (10%) that deemed the NF therapy to be unsafe [27,31].

Injection site reaction was the most commonly observed AE occurring in 61% of patients (*n* = 699) receiving NF by injection. Asthenia, fatigue or weakness in 436 patients (24%), gastrointestinal disturbances in 372 patients (20%), cough in 193 patients (11%) and headache in 173 patients (9%) were also frequently observed. Most common AEs summarized in Table 5 were mild in severity. Serious AE such as death (20), respiratory failure (1), hematemesis (1) and pneumothorax (1) were rarely reported. Finally, one study did not observe any AEs [36].

### 2.5. Relatedness of Adverse Events to the Study Drug in Selected Studies

Nineteen out of twenty studies explicitly stated relatedness of the AE to the NF drug (Table 5). Eight studies (42%) clearly associated the observed AE to the experimental NF drug, while six studies (32%) did not consider the AEs to be related with the NF drug. Moreover, four studies (21%) acknowledged that only some of the observed AEs could have been associated to the NF drug. None of the reported serious AEs were associated with the treatment, but rather with the condition of the patient (i.e., Guillain–Barre syndrome [37], and ALS [38]).

### 2.6. Efficacy Assessment from Selected Studies

Three out of 20 studies failed to report the NF therapy’s efficacy [39,40,41]. Two of these [39,40] conducted safety assessment prior to conducting large scale RCTs with significantly greater sample sizes to derive efficacy assessment [27,28]. The low number of patients and the design of the third study did not permit conclusions to be drawn about the efficacy of the treatment [41].

Authors in 11 [27,31,35,37,40,42,43,44,45,46,47] out of the 17 studies (65%) (Table 6) assessing the efficacy of NF therapy concluded the study drug not to be effective in treating their respective disorders. The remaining six articles [36,38,48,49,50,51] concluded the NF therapy to be effective (Table 3), which will be discussed in the next subsection.

Nine out of 11 trials that considered NF therapy to be ineffective targeted neurodegenerative diseases and failed to demonstrate significantly improved parameters in patients treated with NFs compared to placebo-treated patients. These RCTs attempted to alleviate symptoms of Parkinson’s [31,42], ALS [27,28,35,43], diabetic polyneuropathy [44,45] and Guillain–Barre syndrome [37]. The other two ineffective trials attempted to treat sciatica [46] and retinitis pigmentosa [47].

### 2.7. Influence of the Administration Route and Size of the Effect

Thirteen RCTs opted for a systemic administration of NFs (*n* = 3409 patients). Two of these studies did not report efficacy conclusions [28,39], eight concluded the treatment to be not effective [27,35,37,40,43,44,45,46], while three studies found significant beneficial outcomes [38,48,49] (Table 2). Two of the three latter studies treated ALS and diabetic polyneuropathy [38,48], however, were later contradicted by more recent and larger scale trials [43,44,45]. The other study that deemed systemic NF therapy to be effective examined the effect of systematic administration of CNTF in obese adults [49]. The placebo group’s weight remained the same, while those receiving CNTF showed weight loss of up to 4.1 kg. Three of the four trials that administered NFs locally, considered NF therapies to be effective; these three trials treated non-neurodegenerative diseases (ulcer [36], macular degeneration [50], and sudden deafness [51]).

The size of the effect in the study by Landi et al. compared the prognosis of ulcers of the foot of 36 patients by tracing the perimeter of the wound [36]. The average reduction in pressure ulcer area at six weeks was significantly greater in the NF treatment group than in the conventional topical treatment control group (1.5 times greater reduction). The primary endpoint in the study by Zhang et al. was the change in best-corrected visual acuity one year after treatment [50]. CNTF treatment resulted in improved visual acuity stabilization in the high-dose group (96%) compared with low-dose (83%) and sham (75%) group. The final study that considered NF therapy to be effective applied gelfoam infiltrated with IGF to alleviate sudden deafness [51]. Sixty-six percent of IGF-treated patients showed hearing improvement compared to 53% of the patients treated with conventional steroid dexamethasone. Although statistically significant, the difference in changes in pure-tone average hearing thresholds over time between the two treatment groups was six decibels at best.

## 3. Discussion

In order to foresee the safety and efficacy of NF treatment of the human auditory nerve, we conducted a systematic review of the available literature assessing NF-based treatments for various human diseases, and later addressed its applicability to the auditory nerve. Despite the evident heterogeneity, the results from the reviewed studies can aid in conducting human trials applying NFs to the auditory nerve. The overall results of this review suggests that even if NFs stimulate other cell types in the cochlea or enter the human brain, AEs are not expected to be detrimental. Adverse effects occurred mostly as a result of systemic administration and were generally mild in severity. It should be noted that a search for clinical trials led to the finding of two additional studies using NFs that have not been published. Negative results, with respect to efficacy and/or safety, could be a reason for the lack of publication, since authors and their sponsors may be reluctant to report negative outcomes. The present review highlights that targeted local deliveries of NFs led to favorable safety and efficacy outcomes in three out of four studies [36,50,51]. Therefore, NF administration regimens that successfully target a degenerated neuronal population such as spiral ganglion cells or cochlear synapses may well have promising clinical effects.

### 3.1. Safety and Efficacy of Neurotrophic Therapies of Degenerative Disorders

The present systematic review identified two RCTs that observed AEs serious enough to deem the NF therapy unsafe [27,31]. However, the authors of one of these studies deemed therapy to be safe after refining their application modality [31,42].

The first study monitored disease progression and regression of 730 ALS patients. AEs resulting from subcutaneous CNTF included anorexia, asthenia and cough sufficient enough to limit dosing regimens in patients [27]. The “ALS CNTF Treatment Study Group” revealed that AEs were more severe in patients with in an advanced stage of the disease [27]. This observation suggests that the progression of the disease plays an important role in the tolerability of NF therapies. Following this first ALS trial, many groups attempted to alleviate ALS symptoms using NFs and although efficacy results varied, all concluded the therapy to be safe [28,35,38,40,41,43].

The other RCT that raised safety concerns was the first clinical trial that explicitly investigated the role of NFs in Parkinson’s disease [31]. The application of NFs has been extensively investigated in both rodent and primate models of Parkinson’s disease [26]. Based on these preclinical data, a RCT was conducted to examine the effect of NFs on Parkinsonism in human patients by delivering GDNF intracerebroventricularly [31]. The investigators found no clinical effect at doses that produced adverse effects such as weight loss, paresthesia and hyponatremia. Post mortem analysis in one patient showed no evidence of dopaminergic cell or fiber rescue suggesting that the drug administration failed to reach the dopamine-deficient putamen and substantia nigra. In another attempt made by the same group the drug delivery was well tolerated and therapy was deemed safe. However, outcome parameters did not significantly differ between GDNF- and placebo-treated patients diagnosed with Parkinson’s disease [42].

Similarly, despite promising preclinical research [25], clinical trials with ALS patients repeatedly failed to show beneficial effects of NF therapies [27,28,35,43]. Two studies in the early 1990s demonstrated that CNTF protects motor neurons in cell culture and in vivo rodent model [52,53]. Further findings strengthened the beneficial effect of CNTF in a motor-neuron degeneration mouse model of neuronopathy [21,22]. Based on these and various other preclinical discoveries [25,54], CNTF became the first NF to be clinically investigated for ALS in two clinical trials conducted in 1996 enrolling over 1100 patients altogether [27,28]. Both of those trials reported no marked benefit of NF treatment compared to the placebo-treated patients.

### 3.2. Translation from Animal Studies to Human Trials

Subsequent years of translational research highlight that novel NF therapies particularly for neurodegenerative diseases is challenging. Several factors may account for the difficulty in translating animal studies to a human trial [55]. First, animal models do not meticulously mimic the neurodegenerative processes and rates of disease progression in humans [39]. Therefore, these differences may result in markedly different responses to administered NFs. Second, effective methods of drug delivery targeting specific neuronal populations is challenging. The present review identified that route of administration influenced efficacy outcomes of NF therapies. Therefore, ideal therapies should deliver safe but sufficient concentrations of these proteins at specifically targeted neural tissues containing affected and degenerating neurons, while preventing spread to non-targeted regions [56]. Systemic administration of NFs by subcutaneous injection was effective in only three out of 11 studies included in the present review [38,48,49]. Nonetheless, even when therapies target a specific neural area by intracerebroventricular or intraputamenal administration, no beneficial effect in alleviating symptoms of Parkinson’s disease was found [31,42]. The presence of the blood-brain barrier in the CNS preventing proteins with poor pharmacokinetic properties to cross could explain this inefficacy [20,57]. Pharmacokinetic studies have shown that NFs such as BDNF [57] and CNTF [58] have a short half-life; only 2.9 min after intravenous injection in rodents for CNTF [58] and less than 10 min for BDNF [59]. Third, it remains to be determined whether treatment strategies aiming at a single target will be sufficient to cure multifaceted diseases. The inherent complexity of various diseases in humans may require combinational therapies that target multiple pathways. Fourth, it is presumed that efficacy trends greatly vary among patients with an early versus late onset and progression of diseases. Included studies stated that progression of a disease plays an important role in treatment efficacy [27,28,42]. This is also true for the tolerability of treatment since observed AEs were more severe in patients in an advanced stage of the diseases.

Even if RCTs have not yet provided convincing efficacy outcomes, NFs are still considered as promising drug candidates due to their regenerative or neuroprotective capabilities and are continuously being tested in clinical trials for treating various degenerative diseases. Xiao (2016) reviewed recent clinical trials that have not yet been published [60]. These latest trials explore improved drug delivery methods such as encapsulated cell biodelivery (Alzheimer’s: NCT01163825), continuous brain infusion (supranuclear palsy: NCT00005903), intranasal approach (traumatic brain injury: NCT01212679) and eye drops (corneal keratitis: NCT01756456). Trials are also investigating efficacy of various stages of diseases (Alzheimer’s: NCT02271750; Parkinson’s: NCT01621581). The outcomes of these trials will likely be a valuable addition to the published outcomes reviewed here.

### 3.3. Applicability of NF Therapies to the Auditory Nerve

Treatment of the inner ear with exogenous NFs, such as BDNF or NT-3 has successfully prevented spiral ganglion cell degeneration and improved auditory nerve function in animal models [15,16,17,18,19]. Based on the protective effects demonstrated in these studies, NFs are considered prime candidates for auditory nerve fiber rescue in humans and thus, improve the overall benefits from cochlear implants [2,3,4].

Although highly relevant to local application to the inner ear, AEs directly related to auditory or vestibular function have been scarcely reported. Local administration to the inner ear might logically exacerbate these AEs. Only sporadic incidents of vestibular discomfort have been reported (vertigo, incoordination) [38,49], but not significantly more than in placebo-treated controls. Interestingly, tinnitus was reported to be lower in NF-treated patients than in controls [31,51].

To our knowledge, the RCT attempting to alleviate sudden deafness by Nakagawa et al. in 2014 is the first human trial to apply NFs to the inner ear [51]. The authors attempted to reduce cochlear hair cell damage while most auditory research is focusing on reducing auditory nerve degeneration [7,15,16,17,18,20]. Preserving auditory nerve fibers is particularly interesting when trying to optimize the beneficial effect of cochlear implantation [3,8] or for conditions such as “hidden hearing loss” [5,6,19].

Most RCTs mentioned in the present review opted for a systemic administration or invasive administration directly to the CNS. The present review highlights that targeted deliveries of NFs leads to favorable safety and efficacy outcomes [36,50,51]. The overall results of this review suggests that even if NFs stimulate other cell types in the cochlea or enter the human brain, AEs are not expected to be detrimental as most observed adverse reactions were mild.

## 4. Methods

A systematic review was conducted according to the Preferred Reporting Items for Systematic reviews and Meta-Analysis (PRISMA) guidelines [61].

### 4.1. Search Strategy and Study Selection

A systematic search was executed in Embase (www.elsevier.com), Medline (www.nlm.nih.gov), Cochrane (www.cochranelibrary.com) and Global Health (www.ebscohost.com) electronic databases on 30 July 2015. A search update on 2 March 2016 did not reveal additional eligible articles. The search syntax included relevant synonyms for the terms “neurotrophic factor” and “growth factor” (see Table A1, Appendix A for the full search strategy). The search was restricted to studies published after 1995 to delineate the most recent trends in NF therapies. An additional search was performed in clinicaltrials.gov and EudraCT for studies that were completed but not published.

After removal of duplicates, two authors (AB and VJCK) independently screened titles and abstracts according to predetermined inclusion and exclusion criteria. Articles were considered eligible when they assessed safety and/or efficacy of NF therapies in patients with various diseases. Studies with only healthy subjects were excluded, as they did not assess efficacy, and since safety outcomes in those subjects may not be representative for diseased patients. Only comparative studies reporting original data derived from five or more patients were included. When the same data was presented in more than one publication, the most recent was used for data extraction or the studies were combined to retrieve all outcome measures of interest. All divergence between reviewers was resolved by discussion then consensus.

### 4.2. Quality Assessment of Selected Study

All eligible articles underwent critical appraisal for DoE and RoB performed by two authors (AB and VJCK) using predefined criteria (Table 1 and Table 2). DoE was assessed using 6 criteria: indication for treatment (diagnosis), demographic data (age at treatment), treatment approach (NF used, dose, and administration route), efficacy assessment, safety assessment and follow-up time. RoB was assessed using 6 criteria: randomization, blinding, standardization of treatment, standardization of outcomes, standardization of follow up, and missing data. Table 1 presents the quality assessment results and Table 2 describes the criteria per item for the critical appraisal.

The DoE assessment was scored as high when scores were at least 5 out of a possible 6, as moderate when scores were 4 or 4.5, and as low with scores below 4. The RoB assessment based on the Cochrane Collaboration’s tool for assessing RoB was scored as low when scores were at least 5 out of a possible 6, as moderate when scores were 4 or 4.5, and as high with scores lower than 4. Articles included for data extraction scored: (1) high for DoE and low for RoB; (2) moderate for DoE and low for RoB; or (3) high for DoE and moderate for RoB.

### 4.3. Data Extraction

Descriptive data were extracted by two authors (AB and VJCK) and included study population, diagnosis, administered NF, dosage, administration route, duration of treatment, therapeutic outcome, and AE. The most common AEs were extracted if they occurred in at least 10% of the NF-treated patients per article. The relatedness of the study drug to the reported AE was also considered. Serious AEs were extracted if they occurred at all. An AE was considered serious if it resulted in death, was life threatening, required hospitalization or prolongation of existing inpatients’ hospitalization or resulted in persistent or significant disability or incapacity.

Assessments of the efficacy and safety profile of the NF therapies were based on conclusions drawn by the authors of respective studies.

## 5. Conclusions

This systematic review suggests that the application of NFs is generally safe and well tolerated when administered locally. Since local delivery to the cochlea is feasible, NF treatment of auditory nerve degeneration seems promising.

## Figures and Tables

**Table 1:** Critical appraisal of selected studies reporting patients treated with neurotrophic factor.

Study		Directness of Evidence (DoE)							DoE Score		Risk of Bias (RoB)					RoB Score
	Publication Year	Study Design	Indication for Treatment	Demographic Data	Treatment Approach	Efficacy Outcome Measures	Safety Assessment	Follow-Up		Randomization	Blinding	Standardization (T)	Standardization (O)	Standardization (FU)	Missing Data	
Cedarbaum et al. [39]	1995	RCT	●	◑	●	○	●	◑	**M**	●	●	◑	●	●	●	**L**
Cedarbaum [27]	1996	RCT	●	◑	●	●	◑	●	**H**	●	●	○	●	●	●	**L**
Miller et al. [40]	1996a	RCT	●	◑	●	○	●	◑	**M**	●	◑	◑	●	●	●	**L**
Miller et al. [28]	1996b	RCT	●	◑	●	●	●	◑	**H**	●	◑	◑	●	●	●	**L**
Lai et al. [38]	1997	RCT	●	◑	●	●	●	○	**M**	●	●	●	●	●	●	**L**
Apfel et al. [48]	1998	RCT	●	◑	●	●	●	◑	**H**	●	●	◑	●	●	◑	**L**
Borasia et al. [35]	1998	RCT	●	○	●	●	◑	◑	**M**	●	●	●	●	●	●	**L**
Apfel et al. [44]	2000	RCT	●	◑	●	●	●	○	**M**	●	●	●	●	●	●	**L**
Bensa et al. [37]	2000	RCT	●	●	●	○	●	○	**M**	●	●	●	●	●	◑	**L**
Ochs et al. [41]	2000	RCT	●	◑	●	○	●	◑	**M**	●	●	○	●	●	●	**L**
Wellmer et al. [45]	2001	RCT	●	◑	●	●	●	◑	**H**	●	●	◑	●	●	◑	**L**
Ettinger et al. [49]	2003	RCT	●	◑	●	●	●	●	**H**	●	●	◑	●	●	●	**L**
Nutt et al. [31]	2003	RCT	●	◑	●	●	●	◑	**H**	●	●	◑	●	●	◑	**L**
Landi et al. [36]	2003	RCT	●	●	●	●	○	●	**H**	●	●	●	●	●	●	**L**
Lang et al. [42]	2006	RCT	●	◑	●	●	●	◑	**H**	●	●	●	●	●	●	**L**
Sorenson et al. [43]	2008	RCT	●	◑	●	●	●	●	**H**	●	●	●	●	●	●	**L**
Zhang et al. [50]	2011	RCT	●	◑	●	●	●	○	**M**	●	●	◑	●	●	●	**L**
Birch et al. [47]	2013	RCT	●	◑	●	●	●	●	**H**	●	◑	◑	●	●	●	**L**
Nakagawa et al. [51]	2014	RCT	●	◑	●	●	●	●	**H**	●	●	●	●	●	◑	**L**
Rolan et al. [46]	2015	RCT	●	◑	●	●	●	●	**H**	●	●	◑	●	●	●	**L**
Valk et al.	1996	RCT	●	◑	●	●	○	●	**M**	●	●	◑	●	●	○	**M**
Lambiase et al.	1998	CT	●	●	●	●	○	●	**H**	○	○	◑	●	●	●	**H**
Bonini et al.	2000	CT	●	◑	●	●	○	●	**M**	○	○	●	●	●	●	**M**
Parkman et al.	2003	RCT	●	◑	●	●	●	○	**M**	●	●	○	●	●	◑	**M**
Gill et al. [29]	2003	PCS	●	●	●	●	●	●	**H**	○	○	○	●	●	●	**H**
Patel et al. [30]	2005															
Beck et al.	2005	RCT	●	◑	●	●	○	○	**L**	●	●	◑	●	●	○	**M**
Tuszynski et al.	2005	CT	●	◑	●	●	○	●	**M**	○	○	●	●	●	◑	**H**
Slevin et al.	2005	PCS	●	●	●	●	●	●	**H**	○	○	◑	●	●	●	**H**
Slevin et al.	2006															
Slevin et al.	2007															
Nguyen et al.	2006	RCT	●	◑	●	○	●	◑	**M**	●	○	◑	●	●	●	**M**
Sieving et al.	2006	PCS	●	◑	●	●	●	●	**H**	○	○	●	●	●	○	**H**
Marks et al.	2008	CT	●	●	●	●	●	●	**H**	○	○	◑	●	●	●	**H**
Nguyen et al.	2009	RCT	●	◑	●	○	●	●	**M**	●	○	◑	●	●	●	**M**
Zhou et al.	2009	RCT	●	○	●	●	○	◑	**L**	●	○	○	●	●	●	**M**
Sacca et al.	2011	RCT	●	◑	●	●	●	○	**M**	●	●	○	●	●	◑	**M**
Zein et al.	2014	CT	●	●	●	●	●	●	**H**	○	○	●	●	●	◑	**H**
Chew et al.	2015	CT	●	◑	●	●	●	●	**H**	○	○	◑	●	●	●	**H**
Tan et al.	2015	CT	●	●	●	●	○	○	**M**	○	○	◑	●	●	●	**H**

H: high; M: moderate; and L: low; Grading (● = 1 Point, ◑ = 0.5 Point, ○ = 0 Point).

**Table 2:** Legend critical appraisal. Assessment per item for critical appraisal of selected studies.

Directness of Evidence (DoE)	
**Study design**	CT, clinical trial PCS, prospective case series RCS, retrospective case series RCT, randomized control trial
**Indication for treatment** Diagnosis	Clearly reported, ● Not clearly reported, ○
**Demographic data** Age at treatment	Individually reported, ● Means reported, ◑ Not reported, ○
**Treatment approach** NF used, dosage, route of administration	Reported, ● Not reported, ○
**Efficacy outcome measures** Pre and post treatment assessment	Reported, ● Not reported, ○
**Safety assessment** Quantifiable adverse events per patient If drug was attributed to reported adverse events	Reported per patient or per event, ● Events reported but not quantified, ◑ Not reported, ○
**Follow-up** Duration of follow-up at the end of treatment for all tested individuals	˃2 months, ● <2 months, ◑ not reported, ○
Overall DoE score	High, ≥5 points Moderate, between 4–5 points Low, <4
**Risk of Bias (RoB)**	
**Randomization**	Randomized or concealed, ● Not randomized or concealed, ○
**Blinding**	Blinding of patient, researcher, observer, ● Single blind, ◑ No blinding, ○
**Standardization of treatment**	All patients received the same therapy, ● Different types of NFs or dosage used, ◑ Dosage modified throughout trial, ○
**Standardization of outcome measures**	Identical outcome reports, ● Reported however not standardized, ◑ Not reported, ○
**Standardization of follow up**	Identical follow up for all patients, ● Reported however not standardized, ◑ Not reported, ○
**Missing data**	No missing data; missing data mentioned/quantified and Method of handling described, ● Missing data mentioned in study but method of handling Not described, ◑ Missing data not reported, ○
Overall RoB score	Low, ≥5 points Moderate, between 4–5 points High, <4

Grading (● = 1 Point, ◑ = 0.5 Point, ○ = 0 Point).

**Table 3:** Outcome data extracted from included studies using neurotrophic factors in human trials.

Study	Diagnosis	Total pts (NF Group)	NF	Dose	Administration	Safety Conclusion	Efficacy Conclusion
Cedarbaum et al. (1995) [39]	ALS	57 (43)	CNTF	0.5, 1, 3, 7, 10, 30 µg/kg	Thrice weekly s/c injection per week for 2 weeks	Safe	N/A
Cedarbaum et al. (1996) [27]	ALS	730 (485)	CNTF	15, 30 µg/kg	Thrice weekly s/c injection per week for 9 months	Not safe	Not effective
Miller et al. (1996b) [28]	ALS	44 (33)	CNTF	0.5, 2, 5, 10, 20 µg/kg	Thrice weekly s/c injection for 1 month	Safe (0.5, 2, and 5 µg/kg)	N/A
Miller et al. (1996a) [40]	ALS	483 (360)	CNTF	0.5, 2, 5 µg/kg	Daily s/c injection for 6 months	Safe	Not effective
Lai et al. (1997) [38]	ALS	266 (176)	IGF-I	0.05, 0.1 mg/kg	Twice daily s/c injection for 9 months	Safe	Effective
Apfel et al. (1998) [48]	Diabetic polyneuropathy	250 (168)	NGF	0.1, 0.3 μg/kg	Thrice weekly s/c injection per week for 6 months	Safe	Effective
Borasio et al. (1998) [35]	ALS	183 (124)	IGF-I	0.10 mg/kg	Daily s/c injection for 9 months	Safe	Not effective
Apfel et al. (2000) [44]	Diabetic polyneuropathy	836 (394)	NGF	0.1 μg/kg	Thrice weekly s/c injection per week for 12 months	Safe	Not effective
Bensa et al. (2000) [37]	Guillain–Barre syndrome	10 (6)	BDNF	25 µg/kg	Daily s/c injection for a maximum of 6 months	Safe	Not effective
Ochs et al. (2000) [41]	ALS	25 (20)	BDNF	25, 60, 150, 400, 1000 µg/kg	Daily intrathecal delivery for 3 months	Safe (25, 60, 150 µg/day)	N/A
Wellmer et al. (2001) [45]	Diabetic polyneuropathy	27 (19)	BDNF	25, 100 µg/kg	Daily s/c injection for 3 months	Safe	Not effective
Nutt et al. (2003) [31]	PD	50 (38)	GDNF	150, 361, 559, 1588, 3311 µg	ICV for 8 months	Not safe	Not effective
Ettinger et al. (2003) [49]	Obesity	173 (141)	CNTF	0.3,1, 2 µg/kg	Daily s/c injection for 2–3 months	Safe (0.3, 1 µg/kg)	Effective
Landi et al. (2003) [36]	Pressure ulcer of the foot	36 (18)	NGF	50 µg	Daily topical drop for a maximum of 6 weeks	Safe	Effective
Lang et al. (2006) [42]	PD	34 (17)	GDNF	14 µg	Daily intraputamenal continuous infusion for 6 months	Safe	Not effective
Sorenson et al. (2008) [43]	ALS	302 (150)	IGF-I	0.05 mg/kg	Twice daily s/c injections for 2 years	Safe	Not effective
Zhang et al. (2011) [50]	Macular degeneration	36 (24)	CNTF	5 or 10 ng daily release	Intraocular encapsulated cell implant for 12 months	Safe	Effective
Birch et al. (2013) [47]	Retinitis pigmentosa	266 (133)	CNTF3 CNTF4	5 or 20 ng daily release	Intraocular encapsulated cell implant for 12 months	Safe	Not effective
Nakagawa et al. (2014) [51]	Sudden deafness	118 (60)	IGF-I	10 mg/mL	Intratympanic Gelfoam	Safe	Effective
Rolan et al. (2015) [46]	Unilateral sciatica	48 (36)	GDNF	0.3, 1, 3, 10, 25, 50, 100, 200, 400, 800 μg/kg	i/v or s/c injection of a single dose	Safe	Not effective

N/A: not applicable; ALS: amyotrophic lateral sclerosis; PD: Parkinson’s disease; NF: neurotrophic factor; NGF: nerve growth factor; CNTF: ciliary neurotrophic factor; BDNF: brain derived neurotrophic factor; IGF: insulin-like growth factor; GDNF: glial cell line-derived neurotrophic factor; icv: intracerebroventricular; i/v: intravenous; s/c: subcutaneous.

**Table 4:** Summary of patient characteristic and treatment modality in selected studies.

Characteristics	Included, *n* (%)
*n*, total patients in trials	3974
*n*, patients in NF group	2445 (61.5%)
*n*, patients in placebo group	1529 (38.5%)
**Age at treatment, in years (NF group)**	
Mean ± SD	55.2 ± 10.4
**Diagnosis, *n*, patients**	
ALS	2090 (52.6%)
Diabetic polyneuropathy	1113 (28%)
Retinitis pigmentosa	266 (6.7%)
Obesity	173 (4.4%)
Sudden deafness	118 (3%)
Parkinson’s disease	84 (2.1%)
Sciatica	48 (1.2%)
Macular degeneration	36 (0.9%)
Pressure ulcer of foot	36 (0.9%)
Guillain–Barre syndrome	10 (0.2%)
**NF used, *n*, patients (NF group)**	
CNTF	1219 (49.9%)
NGF	580 (23.7%)
IGF-I	510 (20.9%)
GDNF	91 (3.7%)
BDNF	45 (1.8%)
**Administration route, *n*, patients**	
s/c	3385 (85.2%)
Intraocular encapsulated implant	302 (7.6%)
Intratympanic gelfoam infiltrated	118 (3%)
ICV	50 (1.3%)
Topical	36 (0.9%)
Intraputamenal	34 (0.9%)
Intrathecal	25 (0.6%)
i/v	24 (0.6%)
**Administration type, *n*, patients**	
Systemic	3409 (85.8%)
Local	565 (14.2%)
**Duration of treatment**	
>6 months	2669 (67.2%)
1–6 months	1082 (27.2%)
<1 month	105 (2.6%)
Unknown	118 (3%)

ALS: amyotrophic lateral sclerosis; BDNF: brain derived neurotrophic factor; CNTF: ciliary neurotrophic factor; GDNF: glial cell line-derived neurotrophic factor; icv: intracerebroventricular; IGF: insulin-like growth factor; i/v: intravenous; MSA: multiple system atrophy; N: number; NF: neurotrophic factor; NGF: nerve growth factor; SD: standard deviation; s/c: subcutaneous.

**Table 5:** Summary of safety assessments of neurotrophic factor (NF) based treatment in selected studies.

Adverse Events in Patients Receiving NF, *n*, Events	Included, *n* (%)
*n* of patients receiving NF via injection	1144
injection site pain or reaction	699 (61.1%)
*n* of patients receiving NF	1836
Asthenia, fatigue, weakness	436 (23.7%)
Gastrointestinal disturbances ^a^	372 (20.3%)
Cough	193 (10.5%)
Headache	173 (9.4%)
Mood changes ^b^	141 (7.7%)
Dizziness, vertigo, incoordination	135 (7.4%)
Fever/chills/sweating	134 (7.3%)
Dyspnea/respiratory failure	127 (6.9%)
Weight loss/anorexia	112 (6.1%)
Sensation of warmth/shock/paresthesias	98 (5.3%)
Rhinitis	69 (3.8%)
Ophthalmological symptoms ^c^	68 (2.7%)
Tinnitus	51 (2.8%)
Infection	40 (2.2%)
Pain other than injection site	40 (2.2%)
Dyskinesia	34 (1.9%)
Rash/pruritus	32 (1.7%)
Aural fullness	32 (1.7%)
Hypoglycemia	21 (1.1%)
Peripheral edema/joint swelling/hypertension/IOP	20 (1.1%)
**Relatedness of adverse event to NF drug, *n*, study**	
*n* of studies reporting adverse events	19
Yes	8 (42.1%)
No	6 (31.6%)
Some	4 (21.1%)
N/A	1 (5.3%)
**Overall safety assessment, *n*, study**	
*n* of studies reporting on safety	20
Safe	15 (75%)
Not safe	2 (10%)
Safe for lower doses	3 (15%)

IOP, intraocular pressure; *n*, number; N/A, not applicable; NF, neurotrophic factor. ^a^ Includes: nausea/vomiting/diarrhea/constipation; ^b^ Includes: sleep disturbances, depression, behavioral abnormalities; ^c^ Includes: eye hemorrhage, photopsia, miosis.

**Table 6:** Summary of efficacy assessments of NF based treatment in selected studies.

Overall Efficacy Assessment, *n*, Study	*n* (%)
*n* of studies reporting on efficacy	17
Effective	6 (35.3%)
Not effective	11 (64.7%)

## Appendix A

**Table A1:** Search syntax.

Database	Syntax	
Medline	1	Exp Nerve Growth Factors/
	2	(BDNF or (brain adj3 (neurotrophic* or neuro trophic* or neurotropic* or neuro tropic*))).tw.
	3	(NGF or ((nerve or neurotropic* or neuro tropic* or neurotrophi* or neuro trophi* or neuronotrophi* or neurono trophi* or neurite*) adj3 (outgrowth or growth or factor*)).tw.
	4	1 or 2 or 3
	5	Animals/ not (Animals/ and Humans/)
	6	4 not 5
	7	Exp In Vitro Techniques/
	8	“in vitro”.ti.
	9	7 or 8
	10	6 not 9
	11	Limit 10 to yr=”1995-Current”
	12	Limit 11 to clinical trial, all
Embase		Modeled search strategy for Medline, in title/abstract
Cochrane		Modeled search strategy for Cochrane, in title/abstract
Global health		Modeled search strategy for Global Health, in title/abstract
